# A Protocolised Once a Day Modified Early Warning Score (MEWS) Measurement Is an Appropriate Screening Tool for Major Adverse Events in a General Hospital Population

**DOI:** 10.1371/journal.pone.0160811

**Published:** 2016-08-05

**Authors:** Louise S. van Galen, Casper C. Dijkstra, Jeroen Ludikhuize, Mark H. H. Kramer, Prabath W. B. Nanayakkara

**Affiliations:** 1 Department of Internal Medicine, Section Acute Medicine, VU University Medical Center, Amsterdam, The Netherlands; 2 Department of Anaesthesiology, Academic Medical Center, Amsterdam, the Netherlands; Azienda Ospedaliero Universitaria Careggi, ITALY

## Abstract

**Background:**

The Modified Early Warning Score (MEWS) was developed to timely recognise clinically deteriorating hospitalised patients. However, the ability of the MEWS in predicting serious adverse events (SAEs) in a general hospital population has not been examined prospectively. The aims were to (1) analyse protocol adherence to a MEWS protocol in a real-life setting and (2) to determine the predictive value of protocolised daily MEWS measurement on SAEs: death, cardiac arrests, ICU-admissions and readmissions.

**Methods:**

All adult patients admitted to 6 hospital wards in October and November 2015 were included. MEWS were checked each morning by the research team. For each critical score (MEWS ≥ 3), the clinical staff was inquired about the actions performed. 30-day follow-up for SAEs was performed to compare between patients with and without a critical score.

**Results:**

1053 patients with 3673 vital parameter measurements were included, 200 (19.0%) had a critical score. The protocol adherence was 89.0%. 18.2% of MEWS were calculated wrongly. Patients with critical scores had significant higher rates of unplanned ICU admissions [7.0% vs 1.3%, p < 0.001], in-hospital mortality [6.0% vs 0.8%, p < 0.001], 30-day readmission rates [18.6% vs 10.8%, p < 0.05], and a longer length of stay [15.65 (SD: 15.7 days) vs 6.09 (SD: 6.9), p < 0.001]. Specificity of MEWS related to composite adverse events was 83% with a negative predicting value of 98.1%.

**Conclusions:**

Protocol adherence was high, even though one-third of the critical scores were calculated wrongly. Patients with a MEWS ≥ 3 experienced significantly more adverse events. The negative predictive value of early morning MEWS < 3 was 98.1%, indicating the reliability of this score as a screening tool.

## Introduction

Serious adverse events (SAEs) in hospitalised patients are preceded by signs of clinical deterioration in up to 80% of the patients [1]. Therefore, changes in vital parameters such as pulse rate, respiratory rate, and level of consciousness are often considered as predictors of SAEs such as cardiac arrest, death and unplanned intensive care unit (ICU) admissions [1, 2]. To improve timely detection and treatment of deteriorating patients on nursing wards, rapid response systems (RRSs) have been introduced [3–5]. RRSs consist of two different components: an afferent limb consisting of track and trigger systems (TTS) such as Modified Early Warning Score (MEWS) and an efferent limb, a rapid intervention team (RIT) consisting of trained ICU personnel who will deliver immediate treatment to deteriorating patient at the bedside.

Some studies have demonstrated positive effects of implementing TTSs such as MEWS on patient outcomes [6]. On the basis of these results TTSs have been introduced in many hospitals to increase patient safety [7, 8]. Firstly introduced in 1997 by Morgan et al. the TTS functions as the afferent limb and is designed to detect deterioration early [9]. Since this first introduction multiple early warning bedside monitoring tools have been developed and implemented internationally [10, 11]. These TTSs are used to detect deterioration and call upon a team to monitor and treat patients to prevent further deterioration [12]. In the VU university medical center (VUmc), RRS with an afferent limb consisting of a TTS (MEWS) and an efferent limb consisting of a rapid intervention team (RIT) was introduced a few years ago. Because the afferent limb of the system (RIT) did not function optimally, it was decided to reintroduce the MEWS protocol in 2015 and (re)train the clinical staff aiming to change their mind set and improve protocol adherence.

The effectiveness of a RRS is not only decided by the quality of the RIT but also by an appropriate implementation and use of the TTS such as the MEWS [8, 13]. Unfortunately, very few prospective studies have yet been performed investigating the compliance to any TTS protocol in a real-life setting. In addition, although Smith et al. (2008) demonstrated MEWS as a predictor for clinical outcomes retrospectively, prospective studies investigating the ability of the MEWS to predict relevant clinical outcomes in a general in-hospital population are lacking [14]. In addition, no previous studies have investigated the association between MEWS and the chance of 30-day readmissions. Positive association of MEWS with these endpoints can be used to convince doctors and nurses about the value of MEWS as a prediction tool and thereby increase their protocol adherence.

Therefore, the main aim of this study was to determine the protocol adherence mainly to the afferent limb but also to the efferent limb in a real-life setting. The secondary aims were to investigate the ability of once a day MEWS measurement to predict patient outcomes: in-hospital mortality, hospital length of stay, cardiac arrests, ICU-admissions and 30-day readmissions. Ultimate goal was to provide the hospital staff more insights into the value of the MEWS in predicting outcomes in their own patient population and thereby increase the awareness and protocol adherence.

## Materials and Methods

This prospective study was conducted in a large urban university medical centre (VUmc) with approximately 50,000 admissions per annum in the Netherlands.

### Patient selection

In the 7-week inclusion period from the 8^th^ October until the 30^th^ of November, all adult patients who were in hospital at 08.00 at the date of inclusion on five wards (acute admission unit, general surgery, internal medicine, trauma surgery, vascular surgery/urology/nephrology ward) were included. Due to logistical reasons patients from the pulmonary ward were included from the 1^st^ of November. Patients 18 years and older with at least one overnight stay were included. The Ethics committee of VU University Medical Center Amsterdam, approved the study and necessity for informed consent was waived.

### MEWS protocol in our institution

**Fig 1:**
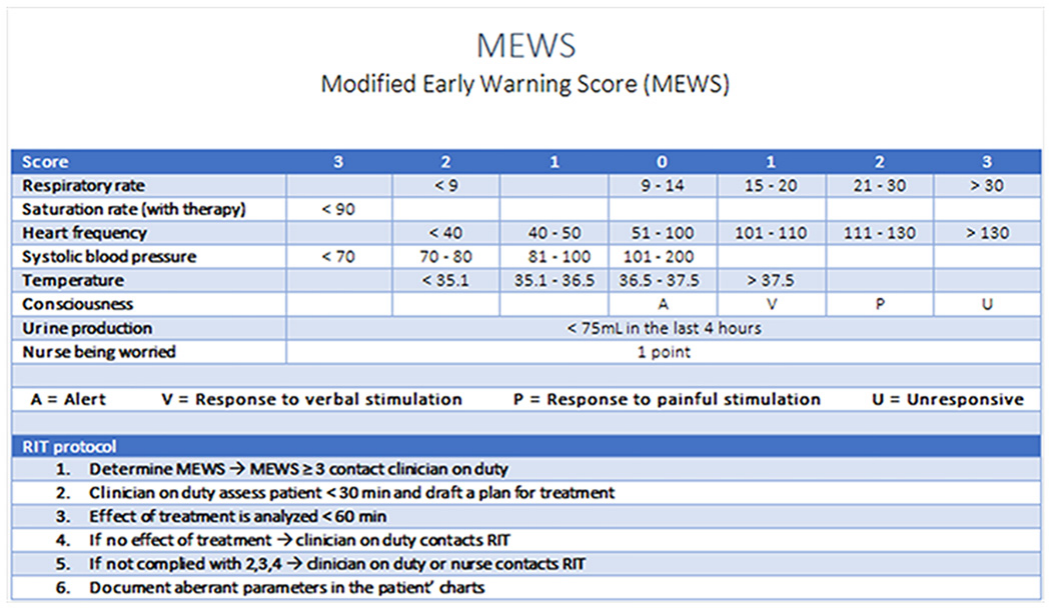
MEWS and protocol in VUmc.

In our hospital all vital parameter measurements are stored in an automatic electronic system. According to the hospital wide protocol, every morning at the end of the nightshift or at the beginning of the dayshift, nurses were requested to determine the MEWS using vital parameter measurements recorded in this electronic system. Although MEWS measurements could be repeated any time during the day on indication by the nurses and doctors, only these early morning scores were used for analysis. The MEWS consists of an easy-to-use algorithm of seven parameters (Fig 1) [15]. The range for the MEWS is between 0 and 19. During the implementation of the protocol staff was trained extensively and the protocol card containing the protocol was distributed. MEWS was calculated by hand and electronically documented in patients’ charts. A total score of 3 or higher was considered as a critical score. Once a patient reaches a critical MEWS (≥ 3) nurses were requested to contact the doctor in charge immediately. The doctor must then assess the patient within 30 minutes and draft a plan for treatment, evaluate this after 60 minutes or call a RIT team. The RIT may also directly be called by the nurses or the doctor at the outset.

### Data collection

Charts of all included patients were checked by the coordinating investigator (CD) to obtain the patients’ MEWS and to determine whether scores were documented and calculated correctly. The MEWS were perceived as documented if MEWS was explicitly reported in nurses charts’. If a vital parameter was not documented in the system, this parameter was considered to be normal. Scores were recalculated by CD using available data in the charts. If a patient had a critical score, charts were examined to find out what actions has been taken, subsequently the nurses and doctors were asked about their actions. If no action was undertaken the investigator inquired the staff about the reasons. If during recalculation a patient had a MEWS of ≥ 3 and this was not explicitly documented by the nurse, nurses were still asked about their actions. However, if a patient had a MEWS of ≥ 3 during recalculation by the CD and this was wrongly calculated and documented by the nurse as a MEWS < 3, no questions were asked. At the end of the inclusion period all answers were categorised. If more than one action was taken, the most serious action was used in the categorisation. For patients who were in hospital for multiple days the highest reached MEWS, labelled as ‘MaxScore’, was taken for predictive analysis.

### Follow up

All patients admitted during the study period were followed up for 30 days after inclusion. In addition, patients were followed up for 30 days after discharge to obtain information about the 30-day unplanned hospital readmission rate. MaxScore per patient was used to perform the predictive analysis of MEWS.

### Statistical analysis

Descriptive characteristics and frequencies were calculated in SPSS version 22.0 (SPSS, Chicago, IL, USA). Categorical outcome measures are presented as frequencies and percentages. Continuous variables are summarised by mean and standard deviation since data was distributed normally. To illustrate the comparison in adverse events between patients who had a MEWS < 3, versus MEWS ≥ 3 a chi-squared test was used. P-values below 0.05 were considered significant.

## Results

### Patient characteristics

A total of 1053 patients were included during the 8-week inclusion period. Table 1 shows patient characteristics. Most patients were admitted to the Acute Medical Unit (n = 408, 38.8%), the least to the general surgery ward (n = 113, 10.7%). The mean age of patients in this cohort was 61.1 (SD 17.6).

**Table 1:** Patient characteristics (N = 1053).

Ward	Patients Number (%)	Male (%)	Mean age (SD)	MaxScore*** Median [range]
**Acute medical Unit**	**408 (38.8)**	**220 (53.9)**	**61.4 (18.9)**	**1.0 [0–9]**
Non-critical score*	365 (89.5)			1.0 [0–2]
Critical score**	43 (10.5)			4.0 [3–9]
**Internal medicine**	**120 (11.4)**	**60 (50.0)**	**66.4 (16.8)**	**2.0 [0–8]**
Non-critical score*	80 (66.7)			2.0 [0–2]
Critical score**	40 (33.3)			3.5 [3–8]
**General surgery**	**113 (10.7)**	**69 (61.1)**	**65.2 (14.5)**	**2.0 [0–8]**
Non-critical score*	**70 (61.9)**			1.0 [0–2]
Critical score**	**43 (38.1)**			4.0 [3–8]
**Vascular/urology/nephrology**	**140 (13.3)**	**92 (65.7)**	**60.1 (14.8)**	**1.0 [0–6]**
Non-critical score*	119 (85.0)			1.0 [0–2]
Critical score**	21 (15.0)			3.0 [3–6]
**Trauma surgery**	**151 (14.3)**	**72 (47.7)**	**53.8 (18.6)**	**1.0 [0–5]**
Non-critical score*	122 (80.2)			1.0 [0–2]
Critical score**	29 (19.2)			3.0 [3–5]
**Pulmonary diseases**	**121 (11.5)**	**56 (46.3)**	**61.6 (14.4)**	**1.0 [0–6]**
Non-critical score*	97 (80.2)			1.0 [0–2]
Critical score**	24 (19.8)			3.5 [3–6]
**Total cohort**	**1053 (100.0)**	**569 (54.0)**	**61.1 (17.6)**	**1.0 [0–9]**
Non-critical score*	853 (81.0)	450 (52.8)	60.5 (17.4)	1.0 [0–2]
Critical score**	200 (19.0)	119 (59.5)	63.8 (18.0)	3.0 [3–9]

* MEWS < 3.

**MEWS ≥ 3.

***MaxScore: Highest reached MEWS for patients who were in hospital for multiple days.

### Measuring and documentation

**Fig 2:**
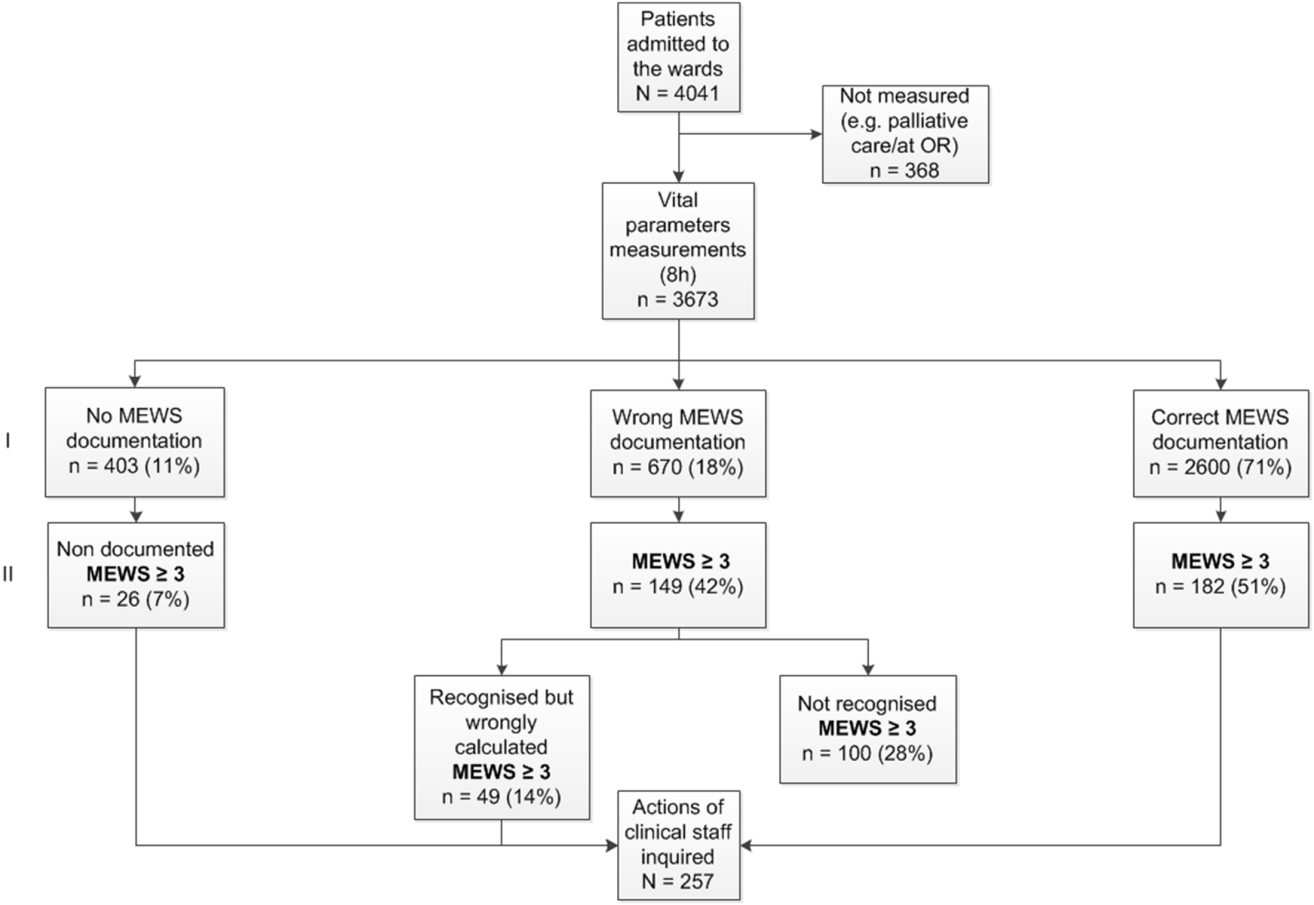
Protocol adherence. Measurement and documentation. Horizontal section I representing all MEWS measurements, regardless of score, Horizontal section II representing MEWS ≥ 3, as recalculated by the coordinating researcher.

There were 4041 patient days where vital parameter measurements could have taken place according to protocol. Fig 2 displays a flowchart of the measurement and documentation. 368 potential measurement moments were missed because these patients were not present on the ward during the time of assessment or because they were in palliative care. This resulted in a total of 3673 morning round measurements in 1053 patients. Of these 3673 vital parameter measurements, 3270 were explicitly documented in nurses’ charts, resulting in a protocol adherence of 89.0%. The investigator recalculated all MEWS using the vital parameters measurements in the charts. The determined MEWS were referred to as recalculated MEWS. We observed a correct calculation in 2600/3673 (70.8%) of the scores in nurses’ charts, 670 (18.2%) scores were calculated incorrectly. The recalculated MEWS were < 3 in 3316 (90.3%) and were ≥ 3 in 357 (9.7%) measurements.

### Actions performed by clinical staff

**Fig 3:**
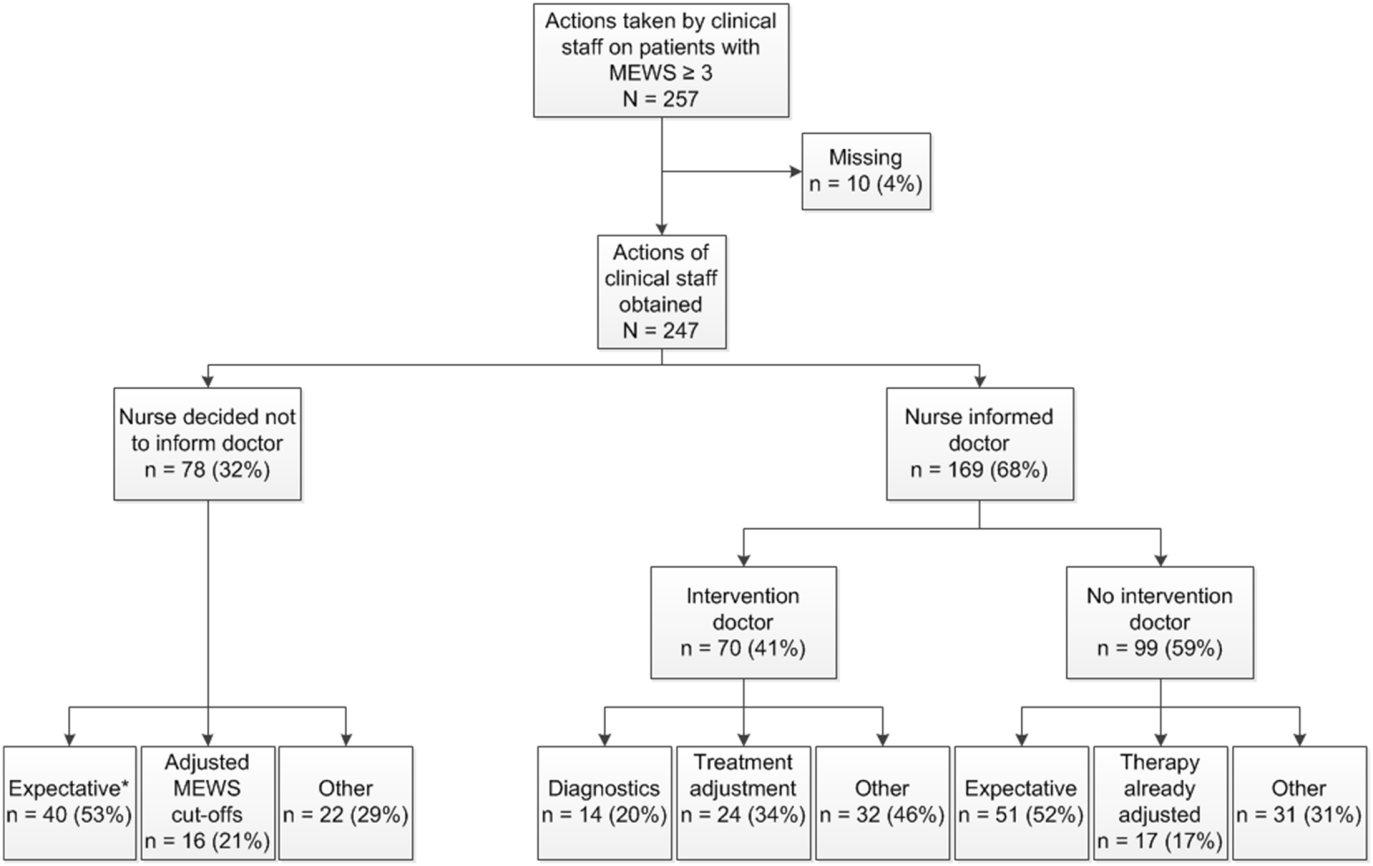
Actions undertaken on patients by clinical staff after critical score reached. N = number of MEWS measurements ≥ 3. *Expectative since this high score is expected as a result of the (known) disease process or the treatment.

In 257 (72.0%) instances in which MEWS ≥ 3 the investigator inquired clinical staff what action they undertook. Fig 3 shows the actions undertaken by hospital staff. In 10 (3.5%) cases no actions could be found in charts and no staff members could answer the questions. Of the remaining 247 cases a doctor was contacted 169 (68.4%) times and 78 (31.6%) times no doctor was contacted. The categorised actions performed are displayed in S1 and S2 Tables. Of the 170 times a doctor was contacted the doctor intervened 70 (41%) times. The main reason for not intervening was that clinical staff did not feel the urge to perform an action since they judged the situation as not alarming enough.

### Patient outcomes

**Fig 4:**
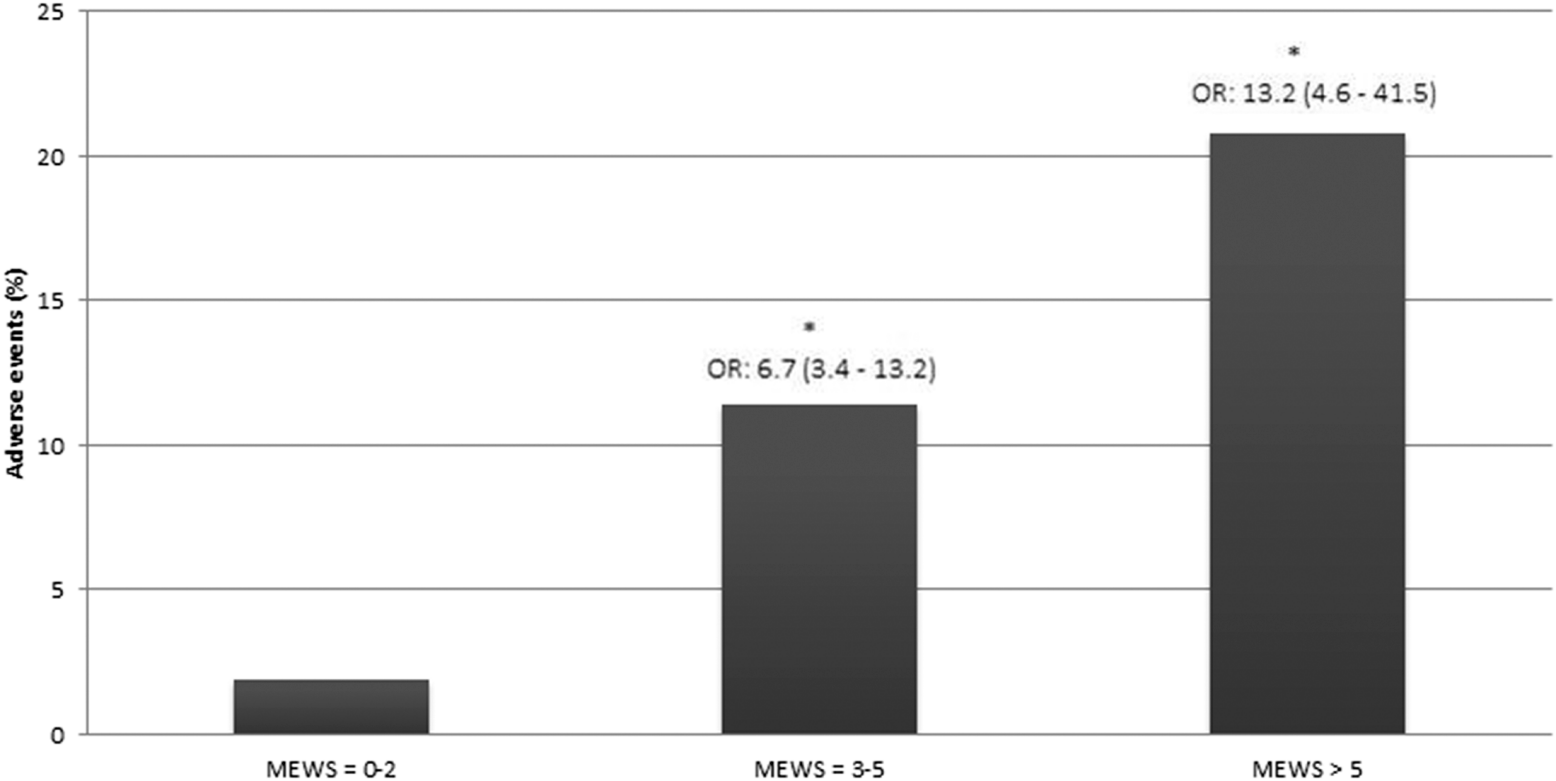
Adverse events compared between MEWS groups. Significant with MEWS < 3 with a p-level of p < 0.001. OR = Odds ratio.

The vital parameters to calculate a MEWS were measured in 1053 patients. Two-hundred patients (19.0%) had a critical score during their hospital stay. The remaining 853 (81%) patients did not have a critical score. Table 2 shows the relation between a critical MEWS and patient outcome. Having a critical score was associated with a higher percentage of unplanned ICU admission [7.0% vs. 1.3%, OR 5.8 (2.6–12.9), p < 0.001], and a higher in-hospital mortality [6.0% vs. 0.8, OR 7.7 (3.0–19.9), p < 0.001]. Also, results show that patients with a critical score had a longer length of stay [15.7 days (SD: 15.7) vs. 6.09 days (SD: 6.9) p < 0.001] and the 30-day readmission rate was higher [18.6% vs. 10.8%, OR 1.9 (1.2–2.9), p < 0.05] than patients without a critical score. Sensitivity for MEWS related to composite adverse events was 61%, specificity 83%, positive predicting value 12.5% and the negative predicting value was 98.1%. MEWS of 3 to 5 show significant more adverse events compared to MEWS below 3. MEWS above 5 show significant more adverse events than MEWS < 3 (p < 0.001) but compared to MEWS 3–5 no significance was reached (p = 0.196). Fig 4 shows patient outcomes compared between different scores.

**Table 2:** Patient outcomes.

	MEWS < 3 n = 853 (81%)	MEWS ≥ 3 n = 200 (19%	Significance	Odds Ratio (95% CI)
**Composite endpoint reached (%)**	16 (1.9)	25 (12.5)	p < 0.001^1^	7.5 (3.9–14.3)
• ICU-admissions	11 (1.3)	14 (7.0)	p < 0.001^2^	5.8 (2.6–12.9)
• In-hospital mortality	7 (0.8)	12 (6.0)	p < 0.001^2^	7.7 (3.0–19.9)
• Resuscitation	0 (0.0)	1 (0.5)	p = 0.190^2^	-
**Readmission (%)**	91 (10.8)	35 (18.6)	p < 0.05^1^	1.9 (1.2–2.9)
**Length of Stay (SD)**	6.09 (6.9)	15.7 (15.7)	p < 0.001^3^	-
**RIT-call (%)**	-	21 (10.5)	-	-

^1^: Pearson Chi-squared.

^2^: Fisher’s Exact test.

^3^: Independent samples t-test.

## Discussion

In this prospective study conducted in a real life setting, we have demonstrated that adherence to the MEWS protocol in our hospital was good (89%). However in some cases (18%) the MEWS was calculated incorrectly because values were not added up properly, influencing the total score. Although, in the majority of the cases the nurse informed the doctor about the critical score an intervention only occurred in one-third of the cases mostly because the situation was judged as not alarming. The MEWS of 3 or higher was a strong predictor of clinical endpoints such as in-hospital mortality, 30-day readmissions, hospital length of stay. In addition, the negative predictive value of MEWS < 3 in this general hospital population was 98.1% indicating the reliability of this score as a screening tool.

The afferent limb is an important component of a RRS, since an effective clinical response depends on early recognition of deterioration [8, 13]. When we implemented the RRS in our hospital a few years ago the afferent limb was implemented without a clear protocol. Therefore the TTS did not function properly. We re-trained the clinical staff and a clear protocol was implemented in 2015. In this protocol nurses were requested to always take a MEWS score in the morning. The main aim of this study was to analyse protocol adherence after this reimplementation. In addition, we aimed to analyse the value of the morning MEWS measurement in predicting clinical outcomes in this general hospital population because this has not been evaluated in a prospective study in a real life setting. The results of this study showed a high protocol adherence with nurses completing MEWS documentation in 89% of the measurements. However, a percentage (18%) of wrongly documented scores were also seen, likely due to wrong calculations in adding up separate MEWS parameters. An important finding was that due to these wrong calculations, a relatively high percentage of critical scores were missed by nurses. Twenty-eight percent of the critical scores, where a doctor was supposed to be alarmed, were not recognised by the nurses. Our study has also shown, that doctors were not contacted in one-third (31%) of the critical scores. When physicians were contacted, they only undertook an action in 28% of the cases. The main reason for not taking action was that staff judged the situation as not alarming. These findings are comparable to Jones et al. (2011) who also found a percentage of 29% [16]. Reasons for these findings, as explained in previous work, are that clinical staff feel the parameter is too rigorous in its cut-offs or the nursing staff estimate the situation as being under control [17, 18]. However, previous work has already demonstrated that changing the critical cut-off to 4 devaluates MEWS as a reliable screening tool [19].

Since creating awareness and emphasising the importance of the MEWS can increase protocol adherence a secondary aim was to validate the MEWS as a predictor for adverse events in our own hospital population. We demonstrate prospectively for the first time in a real-life setting that patients with a MEWS ≥ 3 in one of the morning measurements had an increased risk for an unplanned adverse event than the patients with a MEWS < 3. No significant increase was observed for unplanned resuscitations, likely due to the very low incidence of events. To our knowledge, this is the first study validating this MEWS protocol prospectively in a general in-hospital population in real-life setting. One other study has prospectively validated the value of MEWS in predicting adverse events in a European surgical population. This study was also performed in a real-life setting. Their results are consistent with our findings [20]. In addition, a recent publication in Africa validated the MEWS prospectively in a research setting in low-resource circumstances [21]. They too found that the MEWS was a useful triage tool to identify patients at the greatest risk of experiencing an adverse event. We also demonstrate for the first time that MEWS ≥ 3 is associated with a significantly higher readmission rate within 30 days for a critical score (10.8% vs. 18.6%). Since readmissions are known to increase mortality and are associated with functional decline, it again underlines the importance of the MEWS as a screening tool [22–24].

MEWS as part of the RRT system, was implemented in many Dutch hospitals to potentially increase patient safety [25]. MEWS is a relatively low-cost and convenient bedside monitoring tool, however critical scores can lead to a higher workload for clinical staff. This study, however, again emphasised the clinical importance of recognising patients with a MEWS higher than 3 since these patients are at high risk of developing adverse events. In addition, the negative predictive value of MEWS < 3 was 98.1 underscoring the importance of MEWS as a screening tool. Nevertheless, it is worth mentioning only 7% of the patients in our population with a MEWS ≥ 3 were transferred to the ICU. We do not know how many patients were prevented from ICU admission by early recognition and prompt treatment on the wards.

The strength of this study is its prospective study design in a real world general hospital sample in which 3290 MEWS values were analysed. In addition we personally contacted every nurse who was involved with the MEWS or vital parameter measurements to collect information daily. This is the largest prospective study conducted so far validating MEWS as a screening tool in a general in-hospital (medical and surgical) population [26, 27]. The study was conducted in a single-center which uses one specific MEWS protocol. Therefore, results might not be generalised to hospitals using another EWS protocol. Also, since our aim was to determine clinical relevance of MEWS in daily practice, a real-life hospital situation was studied. As a result, the determined MEWS and not the completeness of the vital parameter set was taken into account. This could possibly under- or overestimate the relation between MEWS and patient outcomes.

## Conclusion

In this prospective study performed in a real-life setting we demonstrated that adherence to the MEWS protocol in our hospital is good (89%). A morning Modified Early Warning Score of 3 or higher was a strong predictor of clinical endpoints such as in-hospital mortality, 30-day readmissions, hospital length of stay. In addition, the negative predictive value of MEWS < 3 in this general hospital population was 98.1% indicating the reliability of this score as a screening tool. Therefore, it is important to keep emphasising the clinical relevance of the MEWS among clinical staff.

## Supporting Information

S1 Dataset Vital parameters and measured MEWS. (XLSX)

S2 Dataset Categorisation actions undertaken at MEWS ≥ 3 by clinical staff. (XLSX)

S3 Dataset Patient outcomes. (XLSX)

S1 Table Actions undertaken on patients by clinical staff after critical score reached. *Since it is part of disease/treatment or patient is familiar with abnormalities.
(DOCX)

S2 Table Categorisation of actions of clinical staff. *Since it is part of disease/treatment or patient is familiar with abnormalities.
(DOCX)
