# Supplementary material for: A Protocolised Once a Day Modified Early Warning Score (MEWS) Measurement Is an Appropriate Screening Tool for Major Adverse Events in a General Hospital Population
Source: PLoS One. 2016 Aug 5;11(8):e0160811. doi: 10.1371/journal.pone.0160811 (PMC4975404; doi:10.1371/journal.pone.0160811)
Supplement: S1 Table — *Since it is part of disease/treatment or patient is familiar with abnormalities. (DOCX) [file pone.0160811.s004.docx]

| **S1. Table**  **Title: Actions undertaken on patients by clinical staff after critical score reached** | | | | | |
| --- | --- | --- | --- | --- | --- |
| **Actions clinical staff at MEWS ≥ 3 (N = 257)** | | | | | |
| **Actions after critical score (nurse)** | **171** | **69%** | **Reasons not to act on critical score (nurse)** | **76** | **31%** |
| 1. Start therapy (no doctor informed) | 2 | 1% | 1. Expectative* | 40 | 53% |
| 1. Contacted doctor immediately | 110 | 64% | 1. Adjusted MEWS cut-off | 16 | 21% |
| 1. Informed doctor during handover within 30 minutes | 53 | 31% | 1. Terminally ill/palliative care | 4 | 5% |
| 1. Start therapy and contacted doctor | 6 | 4% | 1. Nurse forgot to contact doctor | 7 | 9% |
|  |  |  | 1. Unreliable | 7 | 9% |
|  |  |  | 1. MEWS improved since previous measurement | 2 | 3% |
|  |  |  | 1. Unknown | 0 | 0% |
| 1. Missing data | 10 |  | 1. Missing data | 10 |  |
| **Actions after critical score (doctor)** | **70** | **41%** | **Reasons not to act on critical score (doctor)** | **99** | **59%** |
| 1. Start diagnostics | 14 | 20% | 1. Unknown/not clear | 4 | 4% |
| 1. Start/change treatment | 24 | 34% | 1. Doctor too busy | 8 | 8% |
| 1. Start diagnostics and treatment | 8 | 11% | 1. MEWS was positive before | 14 | 14% |
| 1. Consult another specialist | 13 | 19% | 1. Judged as not needed* | 51 | 52% |
| 1. More monitoring | 5 | 7% | 1. Therapy already started | 17 | 17% |
| 1. Consult RIT-team | 6 | 9% | 1. MEWS improved since previous measurement | 1 | 1% |
| 1. No action | 99 |  | 1. Terminally ill patient/palliative care | 4 | 4% |
| 1. Missing data | 10 |  | 1. Missing data | 10 |  |

*Since it is part of disease/treatment or patient is familiar with abnormalities
