# Supplementary material for: A Protocolised Once a Day Modified Early Warning Score (MEWS) Measurement Is an Appropriate Screening Tool for Major Adverse Events in a General Hospital Population
Source: PLoS One. 2016 Aug 5;11(8):e0160811. doi: 10.1371/journal.pone.0160811 (PMC4975404; doi:10.1371/journal.pone.0160811)
Supplement: S2 Table — *Since it is part of disease/treatment or patient is familiar with abnormalities. (DOCX) [file pone.0160811.s005.docx]

S2. Table

Title: Categorisation of actions of clinical staff

| **Examples of actions clinical staff** | | | |
| --- | --- | --- | --- |
| **Action to critical score (nurse)** | **Given answer** | **Reason not to act to critical score (nurse)** | **Given answer** |
| 1. Start therapy | Started supplementary oxygen therapy to which the MEWS improved | 1. Expectative* | Diagnosed COPD which explains higher respiratory rate |
| 1. Contacted doctor |  | 1. Adjusted MEWS cut-off | Accept heart frequency up to 120 bpm as instructed by doctor |
| 1. Informed doctor during handover within 30 minutes |  | 1. Terminally ill/palliative care |  |
| 1. Start therapy and contacted doctor | Started supplementary oxygen therapy already and contacted doctor afterwards | 1. Nurse forgot to contact doctor | Busy due to understaffing |
|  |  | 1. Unreliable | Raised heart frequency after exertion |
|  |  | 1. MEWS improved since previous measurement | In the ED the MEWS was higher, compared with that score the current MEWS has decreased |
|  |  | 1. Unknown |  |
| **Action to critical score (doctor)** | **Given answer** | **Reason not to act to critical score (doctor)** | **Given answer** |
| 1. Start diagnostics | Send patient in for X-thorax | 1. Unknown/not clear |  |
| 1. Start/change treatment | Start Amoxicillin | 1. Doctor too busy | Doctor did not pick up the phone, so MEWS was not handed over |
| 1. Start diagnostics and treatment | Blood culture, X-thorax and start NaCl0,9% infusion | 1. MEWS was positive before | Patient was tachycardic for weeks already so no direct intervention is needed |
| 1. Consult another specialist | Asked cardiologist for advise | 1. Judged as not needed* | Judged patient as not acutely ill |
| 1. More monitoring | Remeasurement in an hour | 1. Therapy already started before | Already planned further diagnostics later that day |
| 1. Consult RIT-team | Patient did not respond to treatment, we decided to contact RIT-team | 1. MEWS improved since previous measurement | Parameters improved compared to the night, so no instant action is necessary |
| 1. No action |  | 1. Terminally ill patient/palliative care |  |

*since it is part of the disease/treatment or patient is familiar with abnormalities
